# GuiaTreeKey, a multi-access electronic key to identify tree genera in French Guiana

**DOI:** 10.3897/phytokeys.68.8707

**Published:** 2016-08-02

**Authors:** Julien Engel, Louise Brousseau, Christopher Baraloto

**Affiliations:** 1 CNRS, UMR AMAP (botAnique et Modélisation de l’Architecture des Plantes et des végétations), Boulevard de la Lironde, TA A-51/PS2, F-34398 Montpellier Cedex 5, France; 2 CNRS, UMR EcoFoG (Ecologie des Forêts de Guyane), Campus Agronomique, BP 316, F-97379 Kourou cedex, France; 3 INRA, UMR EcoFoG (Ecologie des Forêts de Guyane), Campus Agronomique, BP 316, F-97379 Kourou cedex, France; 4 INRA, UR0629 URFM (Ecologie des Forêts Méditerranéennes), Domaine Saint Paul, Site Agroparc CS 40509, 84914 Avignon Cedex 9, France; 5 International Center for Tropical Botany, Department of Biological Sciences, Florida International University, 11200 SW 8th Street, Miami, FL 33199, USA

**Keywords:** Electronic key, trees identification, *Xper*², morphological characters, Neotropics, French Guiana, Amazonia

## Abstract

The tropical rainforest of Amazonia is one of the most species-rich ecosystems on earth, with an estimated 16000 tree species. Due to this high diversity, botanical identification of trees in the Amazon is difficult, even to genus, often requiring the assistance of parataxonomists or taxonomic specialists. Advances in informatics tools offer a promising opportunity to develop user-friendly electronic keys to improve Amazonian tree identification.

Here, we introduce an original multi-access electronic key for the identification of 389 tree genera occurring in French Guiana *terra-firme* forests, based on a set of 79 morphological characters related to vegetative, floral and fruit characters. Its purpose is to help Amazonian tree identification and to support the dissemination of botanical knowledge to non-specialists, including forest workers, students and researchers from other scientific disciplines.

The electronic key is accessible with the free access software *Xper*², and the database is publicly available on figshare: https://figshare.com/s/75d890b7d707e0ffc9bf (doi: 10.6084/m9.figshare.2682550).

## Introduction

The tropical rainforest of Amazonia is one of the most species-rich ecosystems on earth, with an estimated 16000 tree species and often more than 200 species of trees per hectare (ter Steege et al. 2013). Due to this high diversity, the botanical identification of Amazonian trees is very difficult and often requires the consultation of taxonomic specialists. Taxonomists usually specialize in only one or few families or genera (Bacher 2012; Joppa et al. 2011) with few botanical experts, including generalist taxonomists and parataxonomists (Schmiedel et al. 2016), able to identify specimens of various families to the genus level. As a result, the number of specimens incorrectly named or unnamed is still very high in many forest inventories and more widely in the world’s plant collections (Goodwin et al. 2015). Also, traditional dichotomous keys impose constraints on identifying tree samples because they rely on a hierarchical and fixed organization of characters that hampers the identification of a sample when one or several characters are not observed at the time of collection. Moreover, traditional keys often focus on Linnaean characters (flowers and fruits, Rejmánek and Brewer 2001), and only few tools aid the identification of plant species based on vegetative characters (Belhumeur et al. 2008). The identification of sterile samples is therefore difficult even though sterile samples are much more common than fertile ones (the proportion of sterile specimens commonly reaches 90-95%, Aymard et al. 2009; Martinez and Phillips 2000) and taxonomists are often unwilling to review sterile material. Furthermore, printed keys are static and are not frequently revised and republished to reflect taxonomic changes. Here we attempt to modernize botanical identification in Amazonia, by developing a user-friendly electronic key to help tree identification in French Guiana.

French Guiana is a French overseas department of about 85000 km² located in the eastern Guiana shield; it is home to approximately 2000 tree and palm species belonging to 404 genera (updated checklist by Molino et al. 2009). We introduce an original multi-access electronic key for the identification of tree genera occurring in French Guiana *terra-firme* forests based on a set of 79 morphological characters related to vegetative, floral and fruit characters that can be selected in any order. In addition, almost all characters and genera are described and illustrated. Its purpose is to help the identification of tree samples and to disseminate botanical knowledge to non-specialists.

## Taxonomic coverage

The key includes all tree genera occurring in French Guiana *terra-firme* forests with a diameter at breast height (d.b.h) ≥ 10 cm. Monocots (i.e., palms) and tree genera occurring in other habitats (e.g., mangroves, savannas) are excluded. A total of 389 genera belonging to 84 families are treated, see Suppl. material 1: ‘Taxonomic ranks’. The taxonomic validity of genera and families has been checked via the Taxonomic Name Resolution Service (Boyle et al. 2013).

**Figure 1.**
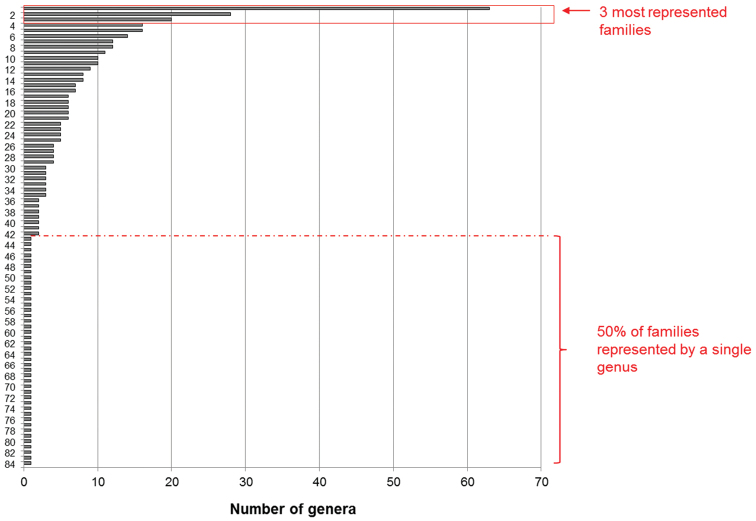
Taxonomic coverage: Number of genera by families: 1 Fabaceae (63); 2 Rubiaceae (27); 3 Euphorbiaceae (20); 4 Annonaceae (16); 5 Malvaceae (16); 6 Lauraceae (14); 7 Apocynaceae (12); 8 Moraceae (12); 9 Myrtaceae (11); 10 Sapindaceae (10); 11 Sapotaceae (10); 12 Rutaceae (9); 13 Chrysobalanaceae (8); 14 Salicaceae (8); 15 Clusiaceae (7); 16 Melastomataceae (7); 17 Anacardiaceae (6); 18 Lecythidaceae (6); 19 Olacaceae (6); 20 Violaceae (6); 21 Burseraceae (5); 22 Capparaceae (5); 23 Humiriaceae (5); 24 Ochnaceae (5); 25 Phyllanthaceae (5); 26 Meliaceae (4); 27 Myristicaceae (4); 28 Urticaceae (4); 29 Vochysiaceae (4); 30 Bignoniaceae (3); 31 Calophyllaceae (3); 32 Celastraceae (3); 33 Malpighiaceae (3); 34 Polygonaceae (3); 35 Proteaceae (3); 36 Achariaceae (2); 37 Bixaceae (2); 38 Combretaceae (2); 39 Linaceae (2); 40 Nyctaginaceae (2); 41 Primulaceae (2); 42 Simaroubaceae (2); 43 Aquifoliaceae (1); 44 Araliaceae (1); 45 Boraginaceae (1); 46 Canellaceae (1); 47 Cannabaceae (1); 48 Cardiopteridaceae (1); 49 Caricaceae (1); 50 Caryocaraceae (1); 51 Dichapetalaceae (1); 52 Ebenaceae (1); 53 Elaeocarpaceae (1); 54 Emmotaceae (1); 55 Erythroxylaceae (1); 56 Goupiaceae (1); 57 Hernandiaceae (1); 58 Hypericaceae (1); 59 Icacinaceae (1); 60 Ixonanthaceae (1); 61 Lacistemataceae (1); 62 Lamiaceae (1); 63 Lepidobotryaceae (1); 64 Loganiaceae (1); 65 Lythraceae (1); 66 Monimiaceae (1); 67 Oleaceae (1); 68 Opiliaceae (1); 69 Pentaphylacaceae (1); 70 Picramniaceae (1); 71 Piperaceae (1); 72 Putranjivaceae (1); 73 Rhabdodendraceae (1); 74 Rhamnaceae (1); 75 Rhizophoraceae (1); 76 Rosaceae (1); 77 Sabiaceae (1); 78 Siparunaceae (1); 79 Solanaceae (1); 80 Stemonuraceae (1); 81 Styracaceae (1); 82 Symplocaceae (1); 83 Ulmaceae (1); 84 Verbenaceae (1).

Figure 1 graphically displays the number of tree genera in each family. The families with the highest number of genera are the Fabaceae (63 genera), Rubiaceae (27 genera) and Euphorbiaceae (20 genera), whereas 42 families (50%) are represented by a single genus.

## Spatial coverage

### General spatial coverage

**Figure 2.**
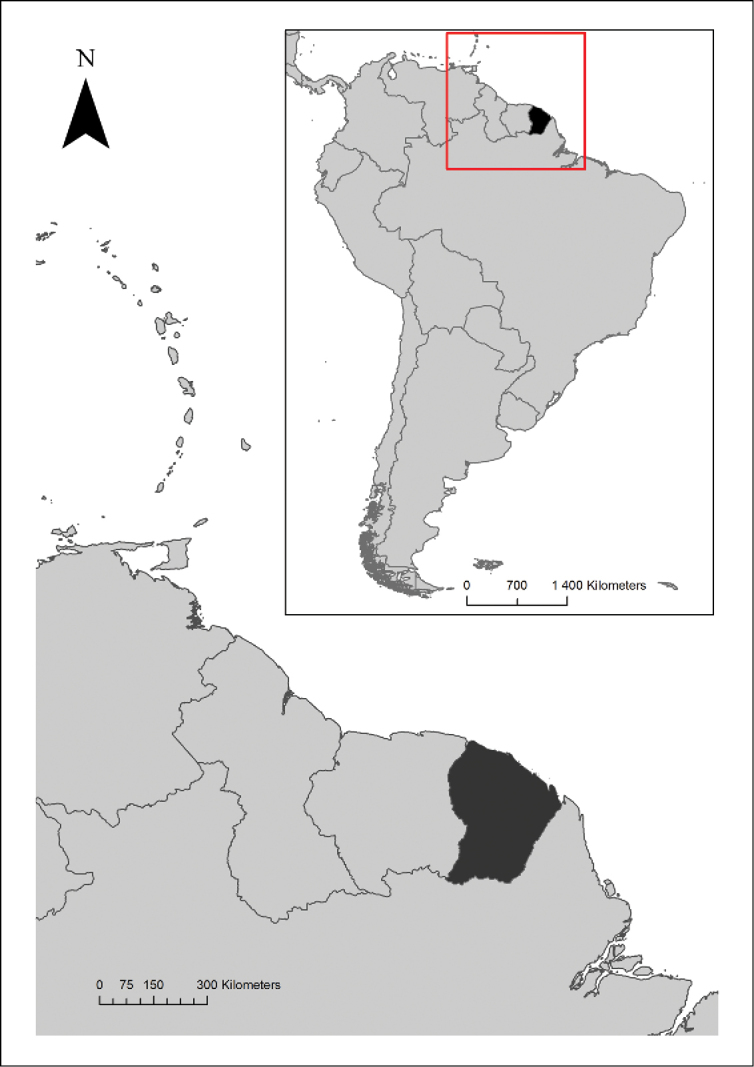
Location of French Guiana in South America.

French Guiana is bordered to the east and south by Brazil and to the west by Suriname (Figure 2). About 90% of the region is covered by evergreen rainforest occurring principally on *terra-firme* soils of granitic or sedimentary origins. The relief is fairly flat with a mean altitude of 140 m and few peaks exceeding 800 m. The climate is equatorial, characterized by a mean annual temperature of 26°C and annual rainfall varying from 2000 mm in the south and west to 4000 mm in the northeast. The rainy season usually occurs between May and August and the dry season between December and January (Guitet et al. 2014).

This key covers French Guiana, but the geographical usefulness is by no means restricted to French Guiana: 99% of the genera included in this key are also present in Brazil (Reflora - Virtual Herbarium. Available at: http://reflora.jbrj.gov.br/reflora/herbarioVirtual/ Accessed on 21/3/2016), more than 90% in Suriname and Guyana, and more than 80% in the Venezuelan states of Amazonas and Bolìvar (Funk et al. 2007). The electronic key may thus also be used in and easily extended to these other regions, although users should keep in mind that these regions also include many other tree genera not covered by this key.

### Coordinates

2°6'42.8"N and 5°45'28.4"N Latitude; 51°38'3.2"W and 54°36'2.7"W Longitude

## Methods

### Electronic key implementation

The electronic key is implemented by a genus × character matrix where the 389 genera are displayed in rows and the 79 characters are displayed in columns, totaling 30731 cells (with less than 3% of missing values). The characters were scored using a comprehensive bibliographic survey of various flora and botanic publications covering the entire region of Amazonia (Acevedo-Rodríguez 2003; Acevedo-Rodríguez 2012; Alford 2009; Alves-Araújo and Alves 2012; Archer and Lombardi 2013; Aymard C and Ireland 2010; Barneby 1989; Barneby et al. 2011; Berg 1972; Berg et al. 1990; Berg and Rosselli 2005; Berry and Wiedenhoeft 2004; Boom 1989; Brandbyge 1986; Chanderbali 2004; 2009; Cornejo 2009; Cowan 1967; Cowan and Lindeman 1989; Da Ribeiro et al. 1999; Da Silva 1986; Da Silva et al. 2010; Daly 1987; De Carvalho-Sobrinho and De Queiroz 2010; De Fraga and Saavedra 2006; Delprete et al. 2010; Díaz 2013; Endress et al. 2014; Esser 2009a; b; Every 2009; 2010; Fernando and Quinn 1995; França 2009; Garcia-Villacorta and Hammel 2004; Gentry 1992; 1993; Graham 2014; Graham and Cavalcanti 2009; Groppo 2010; Groppo et al. 2014; Grose and Olmstead 2007; Guimarães and Monteiro 2010; Gustafsson 2009; Hayden 1990; Hekking 1988; Hiepko 1993; 2000; Hopkins ; Iltis and Cornejo 2011; Jansen-Jacobs 1988; 2007; Jansen-Jacobs and Meijer 1995; Kaastra 1982; Kallunki 1998; Kårehed 2001; Kubitzki and Renner 1982; Landrum and Kawasaki 1997; Maas and Maas-van de Kamer 2012; Maas and Westra 1992; Maas et al. 2003; Madrinan 2004; Marcano-Berti 1998; Mazine and De Faria 2013; McKenna et al. 2011; Melo and França 2009; Mesquita et al. 2009; Michelangeli 2005; Mitchell 1997; Monro and Rodríguez 2009; Morales 2007; Mori et al. 2005; Mori and Prance ; Mori and Prance 1993; Morley 1976; Nee 2001; Pendry 2003; Pennington 1981; 1990; Poppendieck 1981; Prance 1972a; b; 1973; Prance 1986; Prance 2009a; b; c; d; Prance and Mori 1979; Prance and Stace ; Ramos and Lombardi 2009; Redden 2008; Renner and Hausner 2005; Ribeiro et al. 2015; Rodrigues and Goulart de Acevedo Tozzi 2006; Rodrigues and Goulart de Acevedo Tozzi 2008; Rohwer 1993; Romanov et al. 2007; Rudd 1981; Santo et al. 2012; Sastre 2007; Scharf et al. 2008; Schneider and Zizka 2012a; b; Secco 2004; Shepherd and Alverson 1981; Silva 2009; Silverstone-Sopkin 2015; Sleumer 1980; 1984; Sothers et al. 2014; Steyermark et al. 1995; 1997; 1998; 1999; 2001; 2003; 2004; 2005; Teichert et al. 2012; Vasquez Martinez 2013; Westra and Maas 2012; Woodgyer 2009; Wurdack et al. 1993; Zappi 2009).

The characters are grouped into four main sections: ‘leaves’, ‘other vegetative characters’, ‘flowers’, and ‘fruits and seeds’. A substantial proportion of characters (33 of 79) is related to leaves which are almost always observable. In addition, many vegetative characters rarely used in classical dichotomous keys are suggested (e.g., presence of latex, type of trichomes, leaf base venation). Almost all genera and characters are defined and illustrated with more than 9000 photographs (mainly herbarium specimens). Among the 79 characters, 74 are qualitative and 5 are quantitative. Qualitative characters are scored by the presence or absence of the character (e.g., opposite leaves), while quantitative characters are scored by the minimum and maximum number of modalities potentially observable for each genus (e.g., number of calyx segments ranging from three to five): the user may thus select the exact number of modalities observed in the sample.

### Genera-characters matrix file description

The electronic key consists of a genus × character matrix consultable with *Xper*², a software dedicated to taxonomic descriptions and computer-aided identification (Ung et al. 2010). *Xper*² is a user-friendly management system for creating interactive identification keys available on Windows, Mac or Linux in French, English or Spanish versions. It is free software and the botanical keys can be installed locally in order to be used without an internet connection, which is not allowed in the most recent version of the program (*Xper^3^*).

Object name: ‘GuiaTreeKey’

Distribution:

- *Xper*² download page: http://www.infosyslab.fr/lis/?q=en/resources/software/cai/xper2/downloads/last

- ‘GuiaTreeKey’ dataset & User Manual: https://figshare.com/s/75d890b7d707e0ffc9bf (doi: 10.6084/m9.figshare.2682550).

Publication date of data: 23.02.2016

Language: English

Licenses of use: The ‘GuiaTreeKey’ dataset is made available under the Creative Commons Attribution Non-commercial (CC-BY-NC) 4.0 License.

### Software overview and technical features (Figure 3)

**Figure 3.**
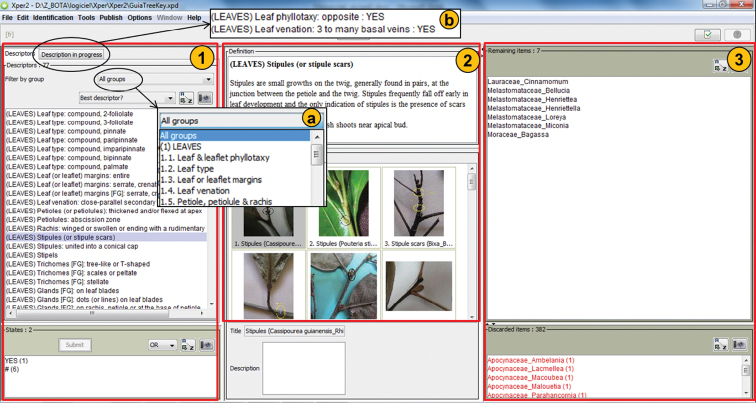
GuiaTreeKey overview.

*PANE 1: Characters box*: In the left pane, the characters are listed and organized by categories and sub-categories (i.e. ‘leaves’, ‘other vegetative characters’, ‘flowers’, and ‘fruits and seeds’, Box a). The user is invited to describe his/her sample using the characters listed in this pane. During the identification process, the user can access a summary of the characters that have been selected (Box b).

*PANE 2*: *Definition and illustration box*: The middle pane displays the definition and illustration of characters and retained genera.

*PANE 3: Results box*: The right pane displays the results in real time. It lists the genera that fit the selected characters. Genera are listed in alphabetic order and they are combined with their family name. A botanical description and photographs of each genus may be displayed in pane 2 by clicking on the genus.

## Examples of identification using GuiaTreeKey

**Figure 4.**
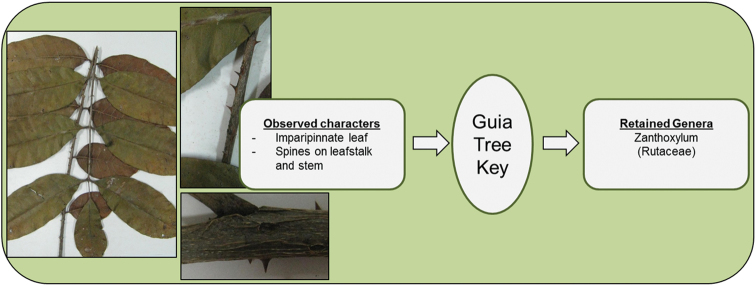
Identification of *Zanthoxylum pentandrum* (Rutaceae).

**Figure 5.**
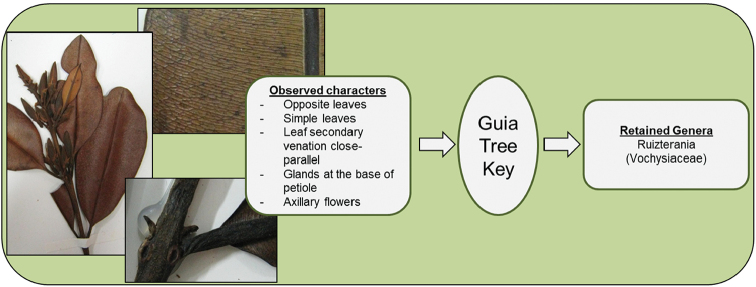
Identification of *Ruizterania ferruginea* (Vochysiaceae).

**Figure 6.**
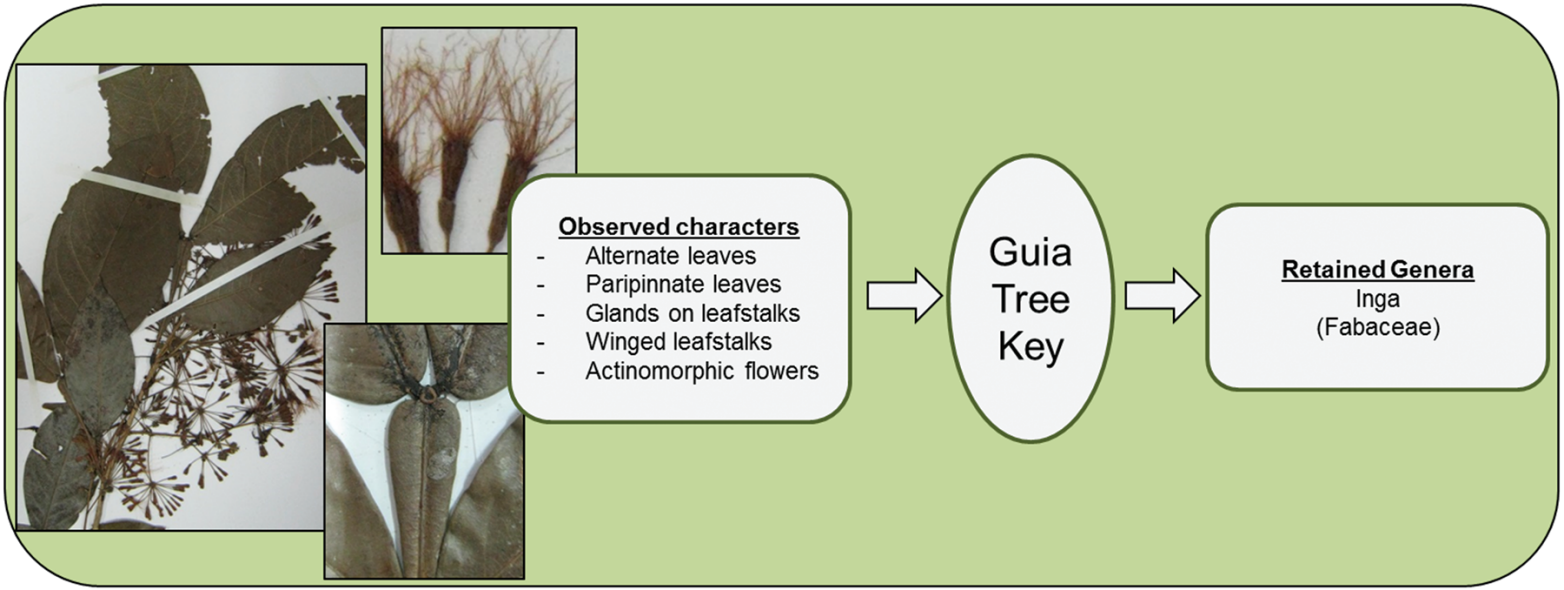
Identification of *Inga umbellifera* (Fabaceae).

**Figure 7.**
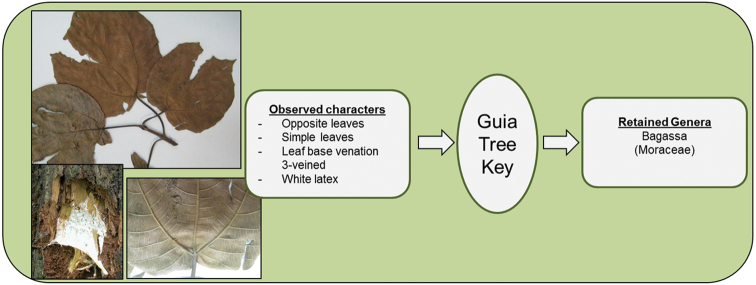
Identification of *Bagassa guianensis* (Moraceae).

In this section, we provide several examples of identification using the electronic key (Figures 4–7).

## Supplementary materials

Supplementary material 1 Taxonomic ranks Data type: list of genera Explanation note: Taxonomic coverage of the electronic key 'GuiaTreeKey': Kingdom, Phylum, Class, Order, Family, Genus This dataset is made available under the Open Database License (http://opendatacommons.org/licenses/odbl/1.0/). The Open Database License (ODbL) is a license agreement intended to allow users to freely share, modify, and use this Dataset while maintaining this same freedom for others, provided that the original source and author(s) are credited.Julien Engel, Louise Brousseau, Christopher Baraloto
