# Supplementary material for: GuiaTreeKey, a multi-access electronic key to identify tree genera in French Guiana
Source: PhytoKeys. 2016 Aug 2;(68):27–44. doi: 10.3897/phytokeys.68.8707 (PMC5029128; doi:10.3897/phytokeys.68.8707)
Supplement: Supplementary material 1 — Taxonomic ranks [file phytokeys-068-027-s001.pdf]

Kingdom: Plantae

Phylum: Tracheophyta

Class: Magnoliopsida

Order: Apiales, Aquifoliales, Boraginales, , Brassicales, Canellales, Caryophyllales, Celastrales, Ericales, Fabales, Garryales, Gentianales, Lamiales, Laurales, Magnoliales, Malpighiales, Malvales, Myrtales, Oxalidales, Picramniales, Piperales, Proteales, Rosales, Santalales, Sapindales, Solanales

Family: Achariaceae, Anacardiaceae, Annonaceae, Apocynaceae, Aquifoliaceae, Araliaceae, Bignoniaceae, Bixaceae, Boraginaceae, Burseraceae, Calophyllaceae, Canellaceae, Cannabaceae, Capparaceae, Cardiopteridaceae, Caricaceae, Caryocaraceae, Celastraceae, Chrysobalanaceae, Clusiaceae, Combretaceae, Dichapetalaceae, Ebenaceae, Elaeocarpaceae, Emmotaceae, Erythroxylaceae, Euphorbiaceae, Fabaceae, Goupiaceae, Hernandiaceae, Humiriaceae, Hypericaceae, Icacinaceae, Ixonanthaceae, Lacistemataceae, Lamiaceae, Lauraceae, Lecythidaceae, Lepidobotryaceae, Linaceae, Loganiaceae, Lythraceae, Malpighiaceae, Malvaceae, Melastomataceae, Meliaceae, Monimiaceae, Moraceae, Myristicaceae, Myrtaceae, Nyctaginaceae, Ochnaceae, Olacaceae, Oleaceae, Opiliaceae, Pentaphylacaceae, Phyllanthaceae, Picramniaceae, Piperaceae, Polygonaceae, Primulaceae, Proteaceae, Putranjivaceae, Rhabdodendraceae, Rhamnaceae, Rhizophoraceae, Rosaceae, Rubiaceae, Rutaceae, Sabiaceae, Salicaceae, Sapindaceae, Sapotaceae, Simaroubaceae, Siparunaceae, Solanaceae, Stemonuraceae, Styracaceae, Symplocaceae, Ulmaceae, Urticaceae, Verbenaceae, Violaceae, Vochysiaceae

Genus: Abarema, Acioa, Acosmium, Agonandra, Aiouea, Albizia, Alchornea, Alchorneopsis, Alexa, Alibertia, Allophylus, Alseis, Amaioua, Amanoa, Ambelania, Ampelocera, Amphirrhox, Anacardium, Anadenanthera, Anaxagorea, Andira, Aniba, Annona, Antonia, Aparisthmium, Apeiba, Aspidosperma, Astronium, Bagassa, Banara, Batesia, Batocarpus, Bauhinia, Beilschmiedia, Bellucia, Bixa, Bocageopsis, Bocoa, Brosimum, Buchenavia, Bunchosia, Byrsonima, Byttneria, Calliandra, Calophyllum, Calycolpus, Calycorectes, Calyptranthes, Campomanesia, Candolleodendron, Capirona, Capparidastrum, Capparis, Caraipa, Carapa, Cardiopetalum, Carpotroche, Caryocar, Caryodendron, Casearia, CassiaCassipourea, Cathedra, Catostemma, Cecropia, Cedrela, Cedrelinga, Ceiba, Chaetocarpus, Chamaecrista, Chaunochiton, Cheiloclinium, Chimarrhis, Chionanthus, Chloroleucon, Chromolucuma, , Chrysobalanus, Chrysochlamys, Chrysophyllum, Cinnamodendron, Cinnamomum, Citharexylum, Clathrotropis, Clusia, Coccoloba, Cochlospermum, Compsoneura, Conceveiba, Conchocarpus, Copaifera, Cordia, Cordiera, Corythophora, Cosmibuena, Couepia, Couma, Couratari, Couroupita, Coussapoa, Coussarea, Coutarea, Crateva, Cremastosperma, Crepidospermum, Croton, Crudia, Cryptocarya, Cupania, Cybianthus, Cymbopetalum, Cynometra, Cyphomandra, Cyrillopsis, Dacryodes, Dendrobangia, Dialium, Dicorynia, Dimorphandra, Dinizia, Diospyros, Diploön, Diplotropis, Dipteryx, Discophora, Dodecastigma, Drypetes, Duguetia, Dulacia, Duroia, Dussia, Ecclinusa, Elaeoluma, Elizabetha, Elvasia, Emmotum, Endlicheria, Enterolobium, Eperua, Ephedranthus, Eriotheca, Erisma, Erythrina, Erythrochiton, Erythroxylum, Eschweilera, Esenbeckia, Eugenia, Euplassa, Exellodendron, Faramea, Ferdinandusa, Ficus, Fusaea, Garcinia, Gaulettia, Geissospermum, Genipa, Gloeospermum, Glycydendron, Goupia, Guapira, Guarea, Guatteria, Guazuma, Guettarda, Guianodendron, Gustavia, Handroanthus, Hasseltia, Hebepetalum, Heisteria, Helicostylis, Henriettea, Henriettella, Hernandia, Herrania, Hevea, Hieronyma, Himatanthus, Hirtella,

Homalium, Hortia, Huberodendron, Humiria, Humiriastrum, Hura, Hydrochorea, Hymenaea, Hymenolobium, Ilex, Inga, Iryanthera, Isertia, Ixora, Jacaranda, Jacaratia, Kubitzkia, Kutchubaea, Lacistema, Lacmellea, Lacunaria, Laetia, Lafoensia, Laxoplumeria, Lecointea, Lecythis, Leonia, Licania, Licaria, Lindackeria, Lonchocarpus, Loreya, Loxopterygium, Luehea, Lueheopsis, Mabea, Macoubea, Macrolobium, Macrosamanea, Mahurea, Malouetia, Manilkara, Maprounea, Maquira, Margaritaria, Marlierea, Martiodendron, Matayba, Matisia, Maytenus, Melicoccus, Mezilaurus, Miconia, Micrandra, Micropholis, Minquartia, Mollinedia, Monopteryx, Moronobea, Mouriri, Myrcia, Myrcianthes, Myrciaria, Myrsine, Naucleopsis, Nectandra, Neea, Neocalyptrocalyx, Neoptychocarpus, Neoraputia, Ocotea, Ophiocaryon, Ormosia, Osteophloeum, Ouratea, Oxandra, Pachira, Pagamea, Palicourea, Paloue, Panopsis, Parahancornia, Paramachaerium, Parinari, Parkia, Pausandra, Paypayrola, Peltogyne, Pentaclethra, Pentascyphus, Pera, Perebea, Persea, Phyllanthus, Picramnia, Pilocarpus, Piper, Platonia, Platymiscium, Plinia, Poecilanthe, Pogonophora, Poraqueiba, Posoqueria, Poulsenia, Pourouma, Pouteria, Pradosia, Preslianthus, Protium, Prunus, Pseudima, Pseudobombax, Pseudolmedia, Pseudopiptadenia, Pseudoxandra, Psidium, Psychotria, Pterocarpus, Ptychopetalum, Qualea, Quararibea, Quiina, Randia, Raputia, Rauvolfia, Recordoxylon, Rhabdodendron, Rhodostemonodaphne, Richeria, Rinorea, Rinoreocarpus, Roucheria, Roupala, Rudgea, Ruizterania, Ruprechtia, Ruptiliocarpon, Ryania, Sacoglottis, Sagotia, Salacia, Sandwithia, Sapium, Sarcaulus, Schefflera, Schistostemon, Scyphonychium, Senna, Sextonia, Simaba, Simarouba, Simira, Siparuna, Sloanea, Sorocea, Spachea, Spirotropis, Spondias, Stachyarrhena, Stenostomum, Sterculia, Stryphnodendron, Styrax, Swartzia, Symphonia, Symplocos, Tabebuia, Tabernaemontana, Tachigali, Talisia, Tapirira, Tapura, Taralea, Terminalia, Ternstroemia, Tetragastris, Tetrameranthus, Theobroma, Thyrsodium, Ticorea, Tocoyena, Toulicia, Touroulia, Tovomita, Trattinnickia, Trema, Trichilia, Trigynaea, Triplaris, Trymatococcus, Unonopsis, Urera, Vantanea, Vatairea, Vataireopsis, Virola, Vismia, Vitex, Vochysia, Votomita, Vouacapoua, Vouarana, Xylopia, Xylosma, Zanthoxylum, Ziziphus, Zollernia, Zygia
